# LncPTEN1, a long non-coding RNA generated from *PTEN*, suppresses lung cancer metastasis through the regulation of EMT progress

**DOI:** 10.1016/j.ncrna.2025.05.011

**Published:** 2025-05-24

**Authors:** Zichu Shen, Ailin Zhong, Chun Zhang, Xiaowei Tang, Xuyang Zhao, Zhiyuan Hou, Hui Liang, Yuxin Yin

**Affiliations:** a Institute of Systems Biomedicine, Department of Pathology, School of Basic Medical Sciences, Beijing Key Laboratory of Tumor Systems Biology, Peking-Tsinghua Center of Life Sciences, Peking University Health Science Center, Beijing, 100191, China; b Department of Breast Surgery, Peking University International Hospital, Beijing, 102206, China; c Department of Pathology, School of Basic Medical Sciences, Peking University Third Hospital, Peking University Health Science Center, Beijing, 100191, China; d Institute of Precision Medicine, Peking University Shenzhen Hospital, Shenzhen, 518036, China

**Keywords:** PTEN, LncRNA, Epithelial-mesenchymal transition, Vimentin, Lung cancer, Tumor metastasis

## Abstract

Lung cancer is among the most frequently observed and lethal malignancies globally, and metastasis represents a critical determinant of patient outcomes. PTEN, a well-established tumor suppressor, has emerged as an important regulator in lung cancer progression. However, the molecular mechanism of *PTEN* gene suppressing lung cancer metastasis lacks deeper exploration. In this research, we identify and characterize LncPTEN1, a novel long non-coding RNA generated from *PTEN* gene. We show that YTHDC1 promotes the alternative splicing of LncPTEN1, resulting in significantly elevated LncPTEN1 expression in normal lung cells. Clinical analyses across multiple patient cohorts demonstrate that low LncPTEN1 expression strongly correlates with poor patient survival and increased metastasis, indicating its potential as a prognostic biomarker. Mechanistically, LncPTEN1 facilitates the interaction between Trim16 and Vimentin, promoting the ubiquitination and proteasomal degradation of Vimentin, thereby suppressing EMT-driven metastasis. The collective evidence from our investigation demonstrates that LncPTEN1 represents a novel tumor-suppressive lncRNA which inhibits lung cancer metastasis through promoting the degradation of Vimentin and inhibiting the EMT progress.

## Highlights

• LncPTEN1 is identified as a novel PTEN-derived long non-coding RNA, uniquely spliced from PTEN exon 1 and intron 1.
• Clinical analyses reveal significant downregulation of LncPTEN1 in lung cancer, correlating with poor patient survival.
• LncPTEN1 facilitates Trim16-mediated Vimentin degradation, thereby suppressing EMT and cancer metastasis.

## Graphical abstract

A schematic illustration depicting the regulatory mechanism of lncPTEN1 in tumor metastasis. In normal cells, lncPTEN1 expression facilitates TRIM16-mediated ubiquitination and degradation of Vimentin through m6A modification regulated by YTHDC1. In tumor cells, decreased lncPTEN1 expression leads to Vimentin accumulation, thereby promoting tumor cell metastasis. This molecular pathway reveals a novel mechanism by which lncPTEN1 functions as a suppressor of tumor metastasis through regulation of the TRIM16-Vimentin axis.

**Figure:**
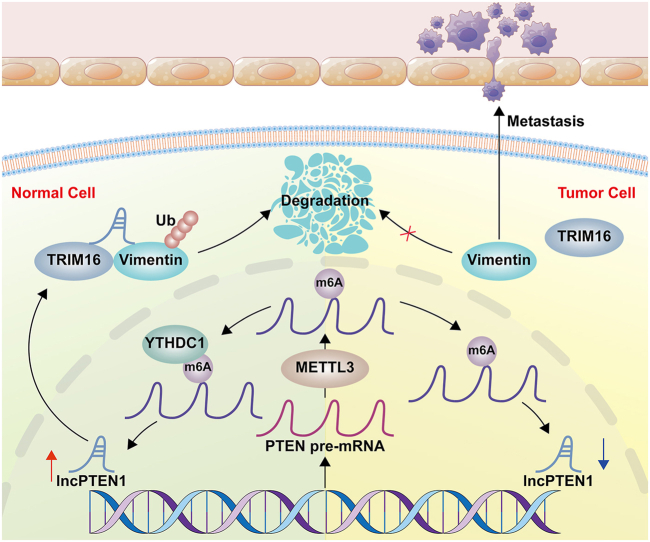

## 1 Introduction

Lung cancer represents the second most diagnosed cancer and the leading cause of cancer mortality worldwide [1,2]. Despite recent therapeutic progress, patient prognosis remains poor, with a 25 % five-year survival rate that falls considerably short of other cancer types [3]. Non-small cell lung cancer is around 80 % of all lung cancer cases, and LUAD is the most aggressive variant [4]. LUAD is characterized by its rapid growth and remarkable metastatic potential, frequently disseminating through the bronchial tree and invading the visceral pleura resulting in early-stage metastasis [5]. The initial phase of metastasis involves epithelial-mesenchymal transition process [6]. Understanding the molecular mechanisms governing EMT-mediated LUAD metastasis is imperative for identifying novel prognostic biomarkers.

Long non-coding RNAs (lncRNAs) are transcripts longer than 200 nucleotides, and absence of coding capacity, serve as essential regulators in cancer development [7,8]. LncRNAs serve multiple roles, including molecular scaffolds, transcriptional regulators, and microRNA sponges, to control gene expression [9,10]. The abnormal expression patterns of lncRNAs play crucial roles in lung cancer progression. TBUR1, a TGF-β-induced lncRNA, enhances LUAD metastasis by stabilizing GRB2 mRNA via hnRNPC interaction, functioning as a critical mediator of EMT [11]. H19 upregulates STAT3 expression through its interaction with miR-17, thereby enhancing the invasion of lung cancer cells [12]. These findings demonstrate that lncRNAs are important regulators in lung cancer development and indicate that exploration of lncRNAs involved in progression of lung cancer may reveal potential therapeutic targets [13,14].

Phosphatase and tensin homolog (PTEN), a key tumor suppressor, ranks as a commonly mutated genomic locus [15]. While PTEN is primarily known for its lipid phosphatase activity within the PI3K/AKT pathway [16], its regulatory network has proven increasingly complex, highlighted by the recent discovery of multiple protein isoforms (PTENα, β, ε) with distinct biological roles [[17], [18], [19]]. In lung cancer, PTEN exerts significant functions in therapeutic responses and disease progression [20]. Furthermore, mutations and dysregulations in the PTEN/PI3K/AKT pathway significantly influence cellular processes critical for lung cancer progression, including cell survival, proliferation, and invasion [21]. Nevertheless, the functional roles of PTEN-associated long non-coding RNAs remain largely unexplored. Investigation of these PTEN-derived transcripts would provide deeper insights into the regulatory complexity of this crucial tumor suppressor and potentially uncover novel biomarkers.

In this study, we identify and characterize a novel long non-coding RNA LncPTEN1, originating from the *PTEN* locus. We reveal that YTHDC1 modulates the processing of LncPTEN1, resulting in its significantly decreased expression in lung cancer compared to normal tissues. Furthermore, bioinformatic analyses indicate that reduced LncPTEN1 levels correlate with poor prognosis in lung cancer patients. Mechanistically, we uncovered that LncPTEN1 functions as a scaffold by facilitating Trim16-mediated ubiquitination and proteolytic degradation of Vimentin. This contributes to the maintain of epithelial characteristics and inhibits lung cancer metastasis. Our findings establish LncPTEN1 as both a novel PTEN-derived tumor suppressor and a potential prognostic biomarker in lung cancer.

## 2 Methods and materials

### 2.1 Clinic samples

A total of 50 matched pairs of lung cancer specimens were from Peking University People's Hospital (Beijing, China) between 2017 and 2020. Following surgical removal, all specimens were immediately frozen in liquid nitrogen for subsequent molecular analyses, including IHC, RNA extraction, and protein isolation. This investigation was approved from Peking University People's Hospital (2020PHB190-01).

### 2.2 Cell lines, antibodies, and reagents

H1299 and A549 were obtained from ATCC. These cells were cultured in RPMI 1640 medium (CORNING), while HEK293 cells were cultured in DMEM (CORNING). Media were enriched with 10 % FBS (HyClone). Withaferin A (WFA) was used as a Vimentin inhibitor (Selleck). The complete information regarding antibodies utilized for Western blot analysis is summarized in SI Table 1.

### 2.3 Plasmids

Expression vectors pCMV-tag-2b, pCDH-GFP, and pLKO.1-puro were obtained from Addgene. The coding sequences of YTHDC1, Trim16, and Vimentin were subsequently inserted into these respective vectors. Homologous recombination was used kit (Yeasen). All oligonucleotide sequences utilized for cloning are documented in SI Table 2.

### 2.4 qRT-PCR

RNA isolation was used TRIzol (Vazyme). RNA purity and concentration used Nanodrop instrument (Thermo Fisher Scientific, USA). First-strand cDNA synthesis used SuperMix (Promega). Real-time PCR used SYBR Green Master Mix (Vazyme) with gene-specific primer pairs in SI Table 2.

### 2.5 Northern blot

Northern blot analysis was performed utilizing DIG Northern Starter Kit (Roche) with protocol modifications. Digoxigenin-labeled probe for lncPTEN1 detection was synthesized *in vitro* from PCR-generated templates. RNA samples (10 μg), with or without RNase R treatment, were separated on 2 % agarose gels and transferred to Hybond-N+ membranes (Beijing Solarbio Science & Technology) via capillary action. The RNA was immobilized by UV cross-linking (200,000 mJ/cm2, 265 nm). Membrane hybridization with biotin-labeled probes proceeded at 68 °C for 6 h. Following blocking, membranes underwent antibody incubation under gentle agitation, three washing steps, and detection solution treatment before X-ray film exposure. GAPDH, analyzed from pre-RNase R treated aliquots, served as the loading control.

### 2.6 Co-immunoprecipitation

For co-immunoprecipitation experiments, protein A/G agarose beads (100 μL) were pre-coupled with anti-Vimentin antibody through 4–8 h incubation. The antibody-conjugated beads underwent two rounds of buffer washing to eliminate excess antibodies. These functionalized beads were subsequently introduced to the protein lysates to capture target antigens. The immunocomplexes were purified by multiple washing steps using 0.1 % NP-40 buffer to minimize non-specific binding.

### 2.7 RNA immunoprecipitation (RIP) assays

The interaction between Vimentin and LncPTEN1 was studied using RNA immunoprecipitation (RIP). Cells lysates were incubated with either a Vimentin-specific antibody or a control IgG for 2 h. Subsequently, beads were added into protein. Post washing, RNA was extracted with TRIzol, treated with DNase.

### 2.8 RNA FISH and IF

RNA FISH assays were conducted following standard protocols [22]. Junction-specific Cy3-labeled LncPTEN1 probes were custom-synthesized (Sangon Biotech, Shanghai, China; probe sequences in SI Table 2). Nuclear counterstaining was achieved using DAPI (Sigma). Specimens were mounted and visualized using a Nikon A1 confocal system.

For protein localization studies, cells grown on coverslips underwent fixation with 4 % paraformaldehyde (15 min), followed by PBS washing steps. Non-specific binding was prevented by blocking with 2 % BSA-PBS solution (1 h). Primary antibody exposure was conducted for 3 h at ambient temperature, followed by secondary antibody incubation (1 h). Nuclear visualization was achieved through DAPI staining (Sigma). Samples were preserved using anti-fade mounting medium before microscopic examination [23]. IF fluorescence intensity quantification was performed using ImageJ. Mean fluorescence intensity was measured for each ROI after background subtraction.

### 2.9 In vitro transcription

The oligonucleotide sequences utilized for *in vitro* RNA synthesis are documented in Supplementary Table 2. RNA transcripts were generated using two distinct systems: biotin-labeled RNA was produced utilizing the T7-Flash Biotin RNA Transcription Kit (Epicenter), while non-modified RNA was synthesized using the Transcript Aid T7 High Yield system (Thermo Scientific).

### 2.10 RNA-protein complex isolation

Cell extracts were prepared by sonication in extraction buffer with protease and RNase. Prior to binding reaction, protein lysates underwent pre-clearing with streptavidin-conjugated magnetic particles to minimize background interference. The biotinylated RNA probes were captured on magnetic beads and exposed to the pre-cleared lysates (4 °C, 6 h). After sequential washing steps, the RNA-associated protein complexes were either subjected to immunoblot analysis or characterized by mass spectrometric methods.

### 2.11 Electrophoretic mobility shift assays

EMSA assays used a commercial kit (Thermo Scientific). The binding reaction mixture contained biotin-labeled lncPTEN1 transcripts (2 nM) and purified Flag-Vimentin protein. Competition assays included excess unlabeled lncPTEN1 (10 mM). The complexes used native polyacrylamide gels, and transferred to nylon membranes, then visualized through streptavidin-HRP conjugate and ECL detection system.

### 2.12 Assessment of 3D spheroid invasion

Multicellular spheroid formation was achieved by seeding single-cell suspensions (2 × 10^4^ cells/well) into non-adherent U-bottom 96-well plates. Following a 72 h incubation period, compact spherical aggregates were obtained. Then, transfer MTSs onto 2 % collagen-coated 96-well plates, allowing the collagen to solidify at 37 °C for 20 min before adding culture medium. Capture spheroid images at baseline and daily for six days post-transfer, maintaining medium freshness bi-daily.

### 2.13 Assessment of cellular motility and invasive potential

Cell migration studies utilized modified Boyden chambers (BD Biosciences). The upper compartment received 50000 cells suspended in serum-deprived medium, while complete medium served as a chemoattractant in the lower chamber. Transmigrated cells were visualized with 0.1 % crystal violet, and quantified across five randomly selected microscopic fields per insert. For invasion analysis, a reconstituted basement membrane matrix (Matrigel) was applied to the upper chamber surface prior to cell introduction.

### 2.14 Xenograft mouse model

Experimental metastasis studies utilized immunodeficient BALB/c nude mice from Vital River Laboratory Animal Technology (Beijing). The pulmonary metastasis model was established in 4-week-old animals via tail vein administration of single-cell suspensions (5 × 10^6^ cells/100 μL PBS). Four weeks post-injection, lung tumors were visualized. Using a Xenogen IVIS Lumina series II system, bioluminescent imaging was performed for a duration of 5 min (Xenogen Corp.).

### 2.15 Data analysis

Functional enrichment analysis of gene sets used clusterProfiler software package [24]. Data visualization and statistical analyses used GraphPad Prism 10.0. Differential expression analysis between tumor and matched non-neoplastic tissues employed paired Student's *t*-tests. Patient survival analysis used Kaplan-Meier methodology.

### Availability of data and materials

Gene expression profiles for Lung Adenocarcinoma (LUAD) were sourced from the UCSC Xena platform, specifically its GDC data repository (accessible via https://gdc.xenahubs.net). Concurrently, YTHDC1 iCLIP data with the accession number GSM2064708 were retrieved from the GEO database [25]. Raw proteomics mass spectrometry data have been submitted to the PRIDE repository (PXD057469) through ProteomeXchange Consortium [26].

## 3 Results

### 3.1 LncPTEN1 is a novel LncRNA generated from PTEN gene and correlates with poor prognosis in lung cancer

**Fig. 1:**
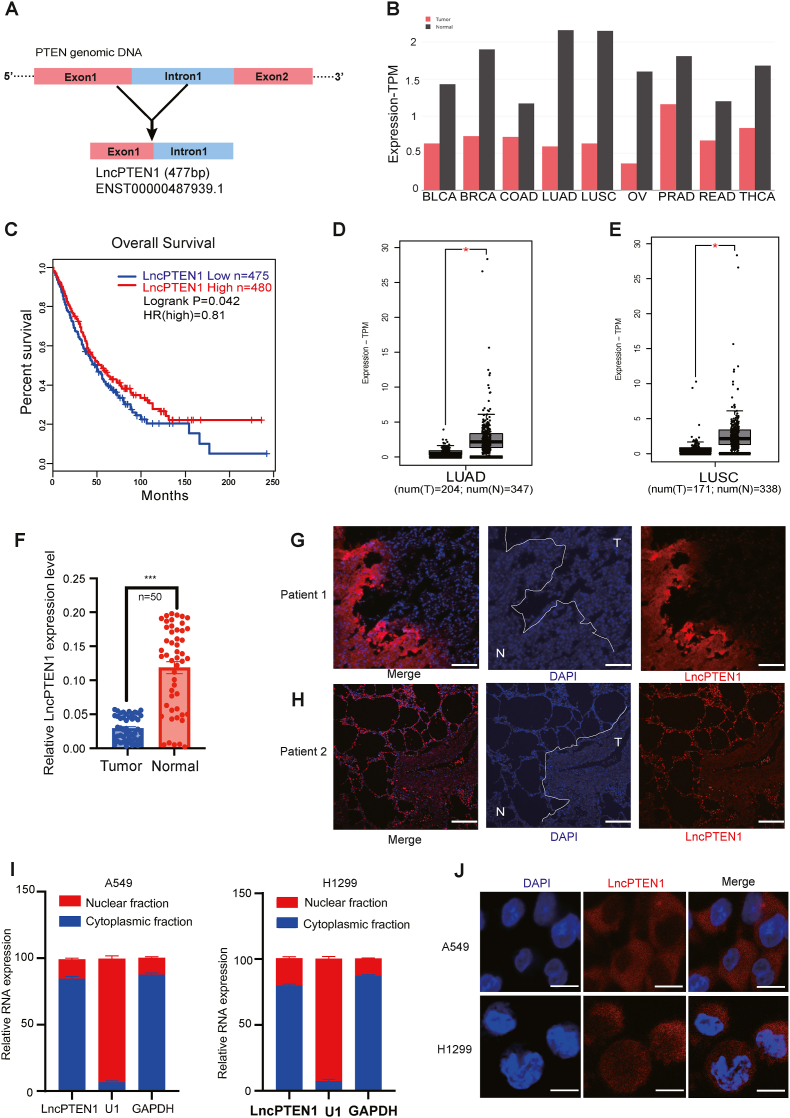
LncPTEN1 is a novel LncRNA generated from *PTEN* gene and associated with poor prognosis in NSCLC. (A) LncPTEN1 genomic structure, showing its derivation from PTEN exon1 and intron1 regions (477 bp, ENST00000487939.1). (B) Pan-cancer analysis of LncPTEN1 expression across multiple cancer types compared to normal tissues based on TCGA data (TPM values). (C) Overall survival of lung cancer patients (n = 475 for low, n = 480 for high; logrank P = 0.042; HR = 0.81). (D–E) LncPTEN1 expression comparison between tumor and normal tissues in LUAD (D) and LUSC (E) cohorts (∗P < 0.05). (F) qRT-PCR analysis of LncPTEN1 expression (n = 50, ∗∗∗P < 0.001). (G–H) RNA FISH results showing LncPTEN1 localization in lung cancer patient specimens. T: tumor tissue; N: adjacent normal tissue. Scale bars, 100 μm. (I) Subcellular fractionation analysis showing the distribution of LncPTEN1 U1 and GAPDH serve as nuclear and cytoplasmic markers, respectively. (J) RNA FISH showing the subcellular localization of LncPTEN1. Scale bars, 20 μm.

Through comprehensive analysis of the Ensembl database, we identified seven potential PTEN-derived LncRNAs (Fig. S1A and B). We further investigated the correlation between PTEN-derived lncRNAs expression and lung cancer survival prognosis using the GEPIA2 database [27]. This analysis revealed that only ENST00000487939.1 exhibited significant correlation with clinical outcomes in lung cancer (Fig. S1C–F). We designated this transcript as LncPTEN1 and selected it for functional and mechanistic investigation (Fig. 1A). To characterize LncPTEN1, our pan-cancer analysis across 33 cancer types using TCGA and GEPIA databases (n = 4740) revealed widespread LncPTEN1 expression in multiple cancers, and demonstrated that its reduced expression was significantly associated with poor patient survival (S2A-B). We then experimentally validated LncPTEN1 through RACE analysis and full-length PCR amplification (Fig. S2C–D). Using primers spanning a 2600 bp intronic region, we confirmed its unique splicing pattern distinct from PTEN mRNA (Fig. S2E). Together, these results established LncPTEN1 as a novel non-coding RNA.

Analysis using the GEPIA2 database demonstrated significant downregulation of LncPTEN1 across multiple cancer types, including BLCA, BRCA, COAD, LUAD, OV, PRAD, and THCA, with particularly pronounced reduction in lung cancer subtypes LUAD and LUSC (Fig. 1B–E). Notably, LncPTEN1 expression exhibited a strong positive correlation with PTEN levels across these cancer types, suggesting a potential functional association (Fig. S3A–D). We substantiated these findings in an independent cohort of 50 paired lung cancer specimens, confirming significant downregulation of LncPTEN1 in lung cancer tissues (Fig. 1F). Moreover, RNA fluorescence in situ hybridization (FISH) of lung cancer tissue sections corroborated these findings, demonstrating markedly reduced LncPTEN1 levels in tumor (Fig. 1G–H). Subcellular localization analysis, employing both nuclear-cytoplasmic fractionation and RNA FISH, consistently revealed predominant cytoplasmic enrichment of LncPTEN1 (Fig. 1I–J). These results not only identify LncPTEN1 as a novel *PTEN*-derived lncRNA but also establish its clinical significance as a potential prognostic indicator in lung cancer.

### 3.2 YTHDC1 promotes LncPTEN1 expression through m6A-dependent splicing regulation

**Fig. 2:**
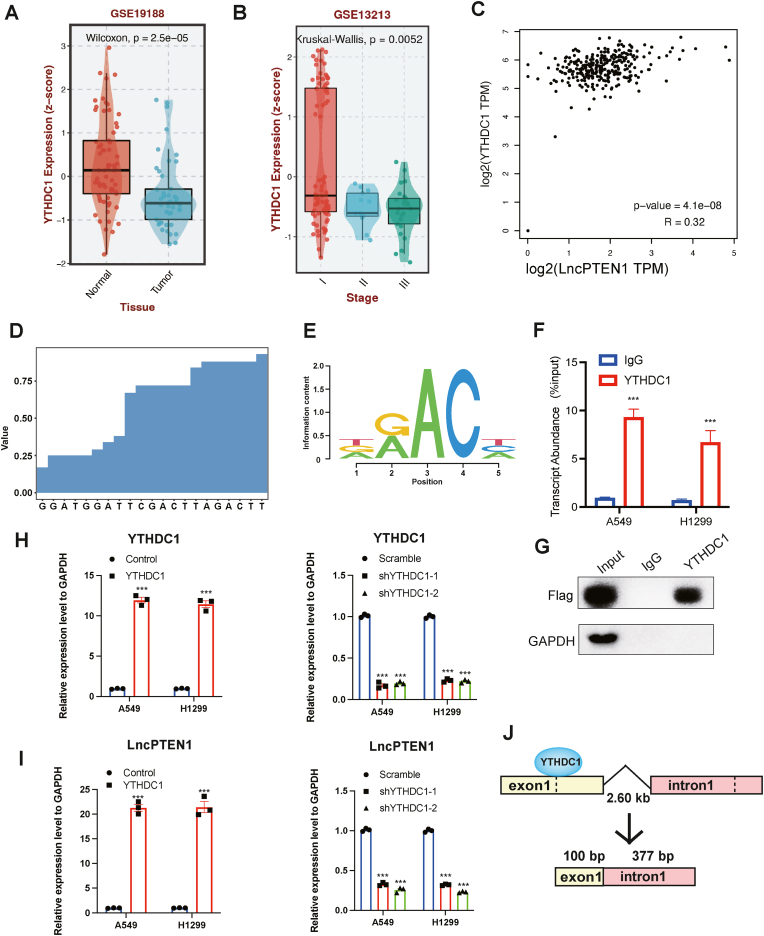
YTHDC1 promotes LncPTEN1 biogenesis through m6A-dependent splicing regulation. (A) Box plot showing differential YTHDC1 expression between normal and tumor tissues from GSE19188 dataset (Wilcoxon, p = 2.5e-05). (B) YTHDC1 expression across different tumor stages in GSE13213 dataset (Kruskal-Wallis, p = 0.0052). (C) Correlation analysis between YTHDC1 and LncPTEN1 expression levels (R = 0.32, p-value = 4.1e-08). (D) YTHDC1 iCLIP Peak Distribution. iCLIP analysis revealing the genomic distribution of YTHDC1 binding sites. (E) Position weight matrix representation of YTHDC1 binding motif. (F–G) RIP assay results showing the enrichment of LncPTEN1 in YTHDC1 pulldown compared to IgG control. (H) qRT-PCR analysis of YTHDC1 expression (∗∗∗P < 0.001). (I) qRT-PCR analysis of LncPTEN1 expression (∗∗∗P < 0.001). (J) Schematic representation of YTHDC1-mediated splicing of LncPTEN1. The diagram illustrates YTHDC1 binding to exon 1, promoting efficient splicing of the transcript.

To elucidate the differential LncPTEN1 expression between lung tumors and normal tissues, we conducted an analysis of TCGA-LUAD gene expression data from the UCSC Xena browser [28]. Notably, YTHDC1 expression was higher in normal tissues and early-stage cancer, exhibiting a positive correlation with LncPTEN1 levels (Fig. 2A–C). Considering the critical roles of m6A modification in RNA metabolism, particularly in splicing, export, stability, and immune regulation [[29], [30], [31]], we propose that m6A-dependent regulation might influence LncPTEN1 biogenesis.

Mechanistically, systematic analysis of m6A modification patterns using the m6AVar database [32] identified m6A modification sites on *PTEN* exon 1 (Fig. S4A). Further investigation of the m6AVar database demonstrated YTHDC1 binding regions coinciding with these m6A modification sites on *PTEN* exon 1 (Fig. 2D). This prediction was confirmed through analysis of YTHDC1 iChIP data [25]. YTHDC1 binding at m6A-modified regions upstream of the LncPTEN1 splicing site in A549 cells, suggesting a direct regulatory relationship (Fig. 2E, Fig. S4B). To substantiate this interaction, we performed RNA immunoprecipitation (RIP) assays, which demonstrated specific binding of YTHDC1 to LncPTEN1 sequences upstream (Fig. 2F and G). YTHDC1 knockdown or overexpression experiments in A549 and H1299 cells both revealed that YTHDC1 levels directly dictated LncPTEN1 expression (Fig. 2H and I). Collectively, these findings establish YTHDC1 as a key regulator that promotes LncPTEN1 biogenesis through m6A-dependent splicing regulation.

### 3.3 LncPTEN1 functions as a novel suppressor of the EMT pathway

**Fig. 3:**
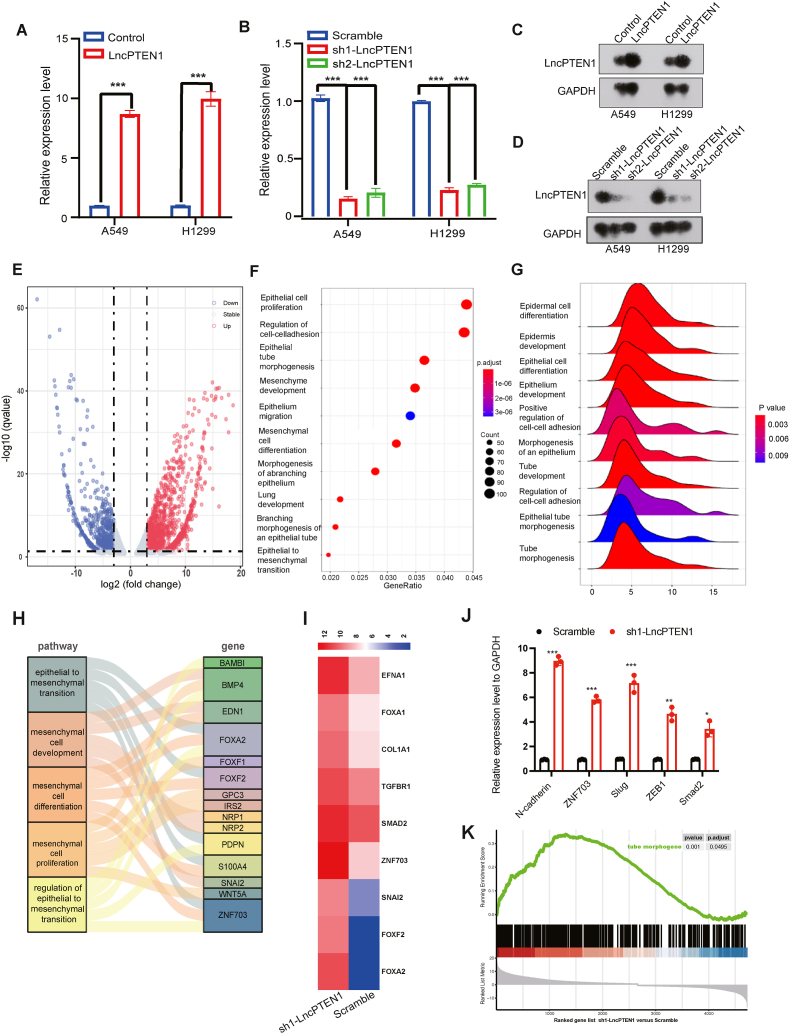
Transcriptome profiling reveals LncPTEN1 as an EMT suppressor. (A) qRT-PCR analysis showing LncPTEN1 overexpression efficiency (∗∗∗P < 0.001). (B) qRT-PCR validation of LncPTEN1 knockdown efficiency using two different shRNAs (∗∗∗P < 0.001). (C) Northern blot analysis confirming LncPTEN1 overexpression. (D) Northern blot validation of LncPTEN1 knockdown. (E) Volcano plot showing differentially expressed genes following LncPTEN1 knockdown (fold change ≥2, ∗P < 0.05). (F) GO analysis showing enriched biological processes. (G) Ridge plot visualization of enriched pathways following LncPTEN1 knockdown. (H) Sankey diagram showing the relationship between EMT-related pathways and their associated genes. (I) Heatmap showing expression changes of key EMT-related transcription factors following LncPTEN1 knockdown. (J) qRT-PCR validation of selected EMT-related genes (∗P < 0.05, ∗∗P < 0.01, ∗∗∗P < 0.001). (K) GSEA showing enrichment of EMT-related pathways in LncPTEN1 knockdown cells.

To investigate the biological function of LncPTEN1, we selected A549 and H1299 cells, which exhibited moderate LncPTEN1 expression (Fig. S3E–F), we established A549 and H1299 stable cell lines with LncPTEN1 overexpression or knockdown. Quantitative PCR (qPCR) analysis confirmed successful modulation of LncPTEN1 levels, with approximately 8-fold increase in overexpression cells and 80 % reduction in knockdown cells (Fig. 3A and B). Northern blot analysis further confirmed the efficacy of both overexpression and knockdown approaches (Fig. 3C and D). Notably, alterations in LncPTEN1 expression did not affect PTEN levels (Fig. S3G and H).

Given the reduced expression of LncPTEN1 in lung cancer, we conducted RNA sequencing on LncPTEN1 knock down cells to identify downstream molecular pathways. Differential expression analysis demonstrated extensive transcriptional reprogramming as visualized in the volcano plot (Fig. 3E). Gene Ontology (GO) analysis revealed significant upregulation of cell adhesion pathways, with notable enrichment in cell-cell junction and cell-matrix interaction signatures (Fig. 3F and G). Strikingly, epithelial-mesenchymal transition (EMT) pathway emerged as one of the most significantly enriched biological processes. This systematic pathway analysis was further confirmed using a Sankey diagram, which revealed connections between EMT-related pathways and their effectors (Fig. 3H). Heatmap analysis demonstrated marked upregulation of key EMT regulators (Fig. 3I). Quantitative PCR (qPCR) confirmed the upregulation of these transcription factors in LncPTEN1 knockdown H1299 cells (Fig. 3J). GSEA also enriched tube morphogenesis pathways (Fig. 3K). Collectively, these findings indicate the activation of EMT pathways in LncPTEN1 knockdown cells (Fig. S5).

### 3.4 LncPTEN1 suppresses the migration and invasion of lung cancer cells through EMT pathway

**Fig. 4:**
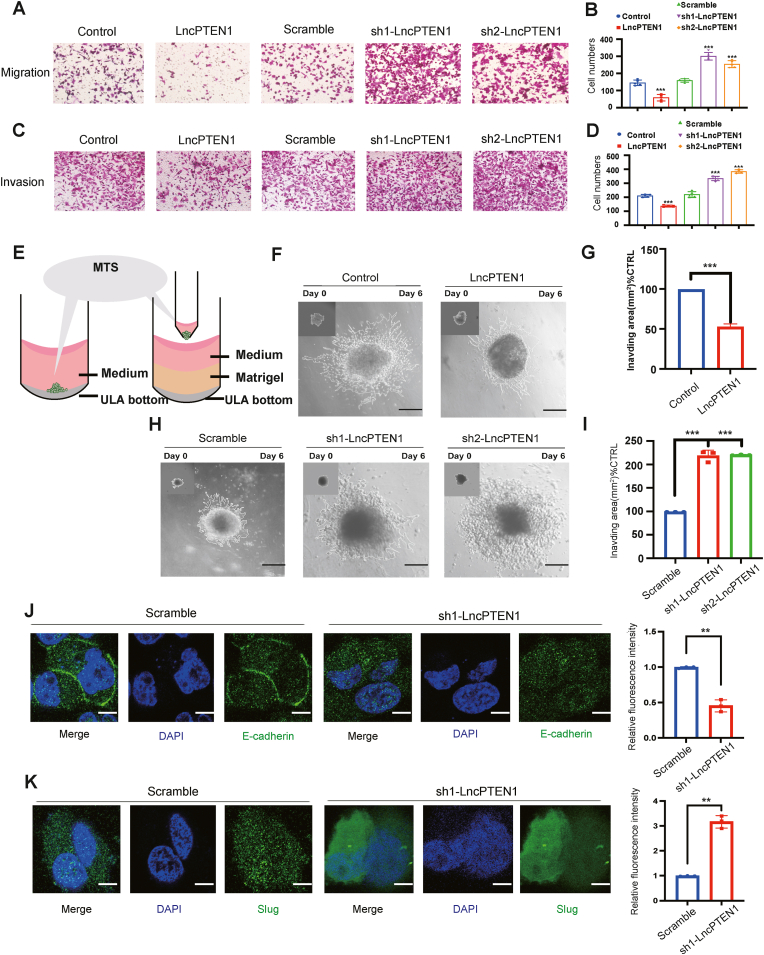
LncPTEN1 inhibits lung cancer cell migration and invasion. (A–B) Representative images of transwell migration assays in A549 cells with LncPTEN1 overexpression or knockdown. Scale bar, 100 μm. ∗∗∗P < 0.001. (C–D) Representative images of matrigel invasion assays in A549 cells. Scale bar, 100 μm. ∗∗∗P < 0.001. (E) Schematic illustration of the three-dimensional spheroid invasion assay using ultra-low attachment (ULA) plates and matrigel matrix. (F–G) Images of control and LncPTEN1-overexpressing H1299 spheroids at day 0 and day 6. Scale bar, 200 μm. Insets show initial spheroid morphology. Quantification of spheroid invasion area from (F), ∗∗∗P < 0.001. (H–I) Images of spheroid invasion in scramble control and LncPTEN1-knockdown H1299 cells. Scale bar, 200 μm. Quantification of spheroid invasion area from (H), ∗∗∗P < 0.001. (J) Immunofluorescence staining showing E-cadherin (green) localization and expression in H1299 cells with scramble control or LncPTEN1 knockdown. Nuclei were counterstained with DAPI (blue). Scale bar, 20 μm. Fluorescence intensity was normalized to the control group, ∗∗P < 0.01. (K) Immunofluorescence staining showing slug (green) localization and expression in H1299 cells with scramble control or LncPTEN1 knockdown. Nuclei were counterstained with DAPI (blue). Scale bar, 20 μm. Fluorescence intensity was normalized to the control group, ∗∗P < 0.01.

To elucidate the regulation of the EMT pathway by LncPTEN1, we assessed its impact on metastatic properties. Transwell migration assays revealed that LncPTEN1 overexpression significantly suppressed cell motility, while its knockdown enhanced migration in H1299 cells (Fig. 4A and B). Matrigel invasion assays demonstrated consistent results, with LncPTEN1 overexpression reducing invasive capacity and its knockdown promoting invasion (Fig. 4C and D). To more accurately construct the three-dimensional tumor microenvironment, we established a spheroid invasion model using ultra-low attachment plates and matrigel matrix (Fig. 4E). After six days, the spheroid area showed that LncPTEN1 overexpression significantly reduced invasive outgrowth (Fig. 4F and G), while its depletion promoted aggressive invasion into the surrounding matrix (Fig. 4H and I). Additionally, IF analysis revealed that LncPTEN1 knockdown disrupted E-cadherin membrane localization and reduced its expression, while promoting the expression and nuclear translocation of Slug (Fig. 4J and K). These molecular alterations were functionally coupled with enhanced cellular migration and invasion capabilities, establishing LncPTEN1 as a critical regulator of the EMT program.

**Fig. 5:**
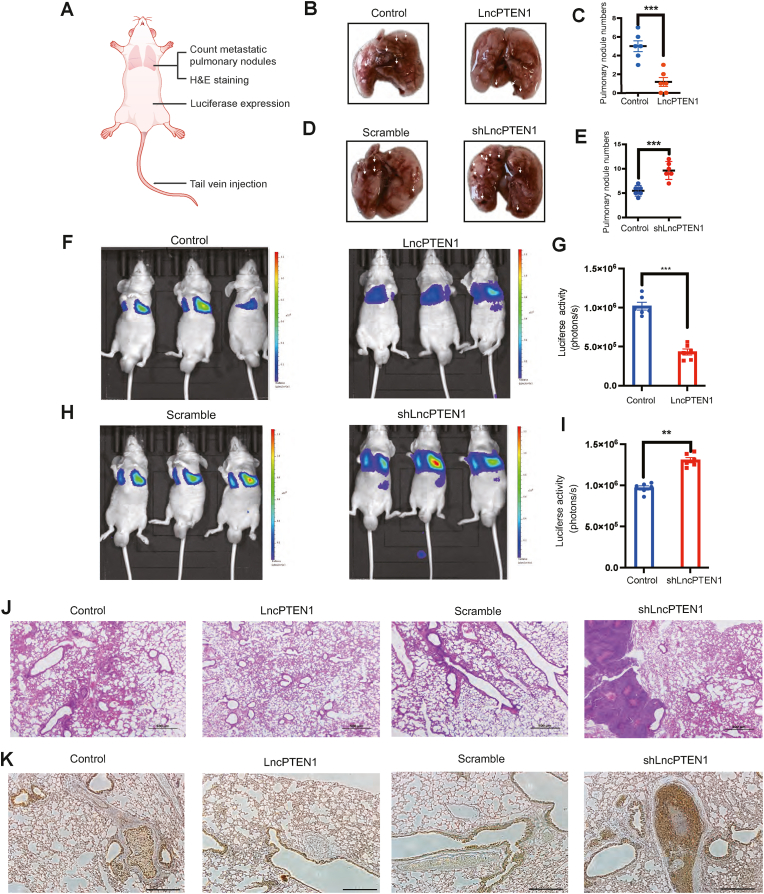
LncPTEN1 suppresses lung cancer metastasis *in vivo*. (A) Schematic of metastasis model using tail vein injection. (B,D) Representative images of mice lungs injected with indicated cells (n = 6 per group). (C,E) Quantification of visible metastatic nodules from (B,D). Data presented as mean ± SD. ∗∗∗P < 0.001. (F,H) Representative bioluminescence images of mice at 4 weeks post-injection. (G,I) Quantification of luciferase activity from (F,H). ∗∗P < 0.01, ∗∗∗P < 0.001. (J) H&E staining of lung sections showing metastatic lesions. Scale bar, 500 μm. (K) Representative immunohistochemical staining of Slug protein in lung cancer tissue sections. Scale bar, 200 μm.

To explore LncPTEN1 functions *in vivo*, we established a lung colonization model by tail vein injection lung cancer cells with differential LncPTEN1 expression into nude mice. Subsequently, we quantified lung nodules, traced fluorescently-labeled cells, and examined histological sections of pulmonary tissue (Fig. 5A). In our study, mice injected with LncPTEN1-overexpressed cells exhibited a reduced number of metastatic lung nodules compared to the control group (Fig. 5B and C). Conversely, LncPTEN1 knockdown increased the number of metastatic nodules (Fig. 5D and E). Furthermore, the bioluminescent signal intensity from the lungs of mice with cells overexpressing LncPTEN1 was significantly diminished relative to that of the control mice, indicating a lower metastatic burden (Fig. 5F and G). While LncPTEN1 knockdown accelerated metastatic spread (Fig. 5H and I). Moreover, histological examination of pulmonary tissue sections with H&E staining revealed evident metastatic nodules in control lungs, while lungs from the LncPTEN1 overexpression group displayed markedly reduced tumor burden and severity of malignant lesions (Fig. 5J). Immunohistochemical analysis revealed increased Slug staining in metastatic lesions of LncPTEN1 knockdown tumors compared to control groups, while LncPTEN1 overexpression reduced Slug expression in metastasis (Fig. 5K). These findings demonstrate that LncPTEN1 suppresses lung cancer metastasis through regulation of the EMT pathway.

### 3.5 LncPTEN1 regulates EMT pathway through interaction with vimentin

**Fig. 6:**
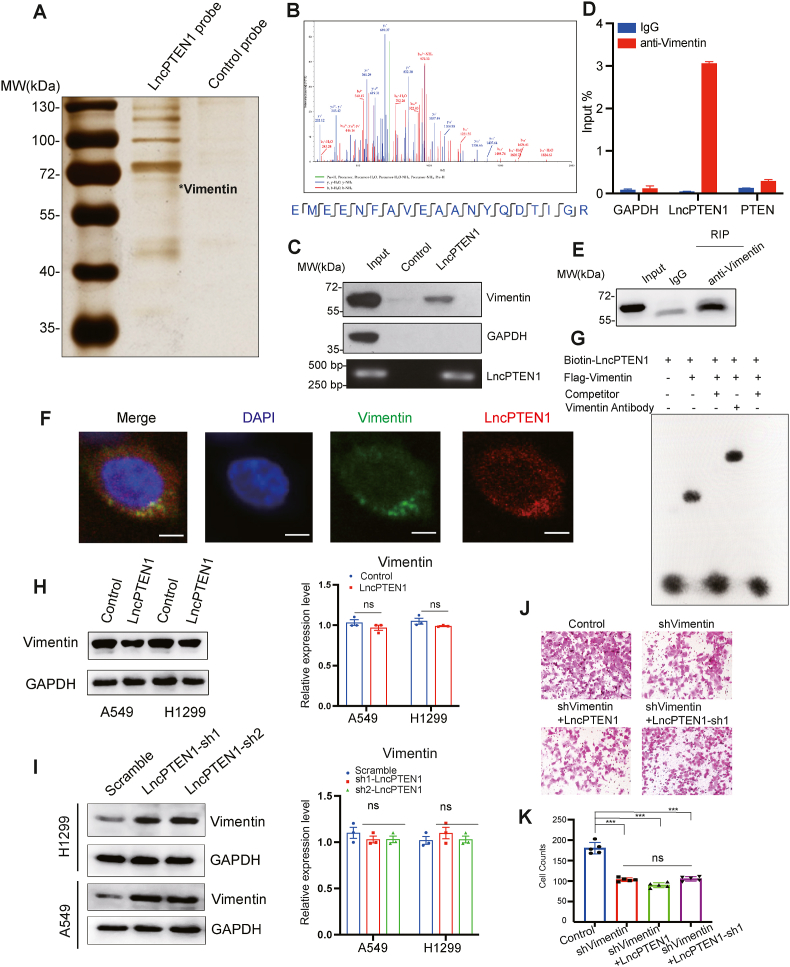
LncPTEN1 suppresses the EMT pathway through interaction with Vimentin. (A) RNA pull-down assay followed by silver staining showing proteins interacting with biotinylated LncPTEN1. ∗indicates Vimentin band. (B) Mass spectrometry analysis of LncPTEN1-interacting proteins. (C) Western blot validation of Vimentin interaction with LncPTEN1 in RNA pull-down assay. GAPDH serves as negative control. (D) RIP-qPCR showing enrichment of LncPTEN1 in Vimentin immunoprecipitates. GAPDH and PTEN serve as controls. (E) Western blot confirming Vimentin immunoprecipitation efficiency. (F) RNA FISH and IF co-staining showing colocalization of LncPTEN1 (red) and Vimentin (green) in lung cancer cells. Scale bars, 20 μm. (G) Biotin pull-down competition assay demonstrating specific interaction between LncPTEN1 and Flag-Vimentin. (H) Western blot and qRT-PCR analyses showing Vimentin expression levels in control and LncPTEN1-overexpressing A549 and H1299 cells (ns: not significant). (I) Western blot and qRT-PCR analyses showing Vimentin expression levels following LncPTEN1 knockdown in A549 and H1299 cells (ns: not significant). (J) Representative images of transwell invasion assay showing the effects of Vimentin knockdown combined with either LncPTEN1 overexpression or knockdown in H1299 cells. Scale bar, 100 μm. (K) Quantification of H1299 cells from (J) (∗∗∗P < 0.001, ns: not significant).

To uncover the molecular mechanism of LncPTEN1 in modulating tumor progression, we conducted RNA pull-down and proteome analysis. Silver staining demonstrated the presence of several specific protein bands enriched in the LncPTEN1 pull-down fraction, with prominent signals between 55 and 70 kDa (Fig. 6A). Proteomic profiling identified Vimentin, a key EMT marker, as the most significantly enriched protein based on both peptide abundance and specificity scores (Fig. 6B, Fig. S5A–C). The physical binding between LncPTEN1 and Vimentin was verified through RNA affinity purification and RIP analyses (Fig. 6C–E). The co-localization of LncPTEN1 with Vimentin was revealed by IF analysis (Fig. 6F). EMSA assay further verified that LncPTEN1 direct interact with Vimentin (Fig. 6G). Mechanistically, we found that LncPTEN1 overexpression significantly decreased Vimentin protein levels without affecting its mRNA expression, while LncPTEN1 knockdown elevated Vimentin protein abundance (Fig. 6H and I). To establish the functional significance of this interaction, we constructed Vimentin knockdown H1299 cells. Vimentin knockdown reduced cell migration in transwell assays. Furthermore, in Vimentin knockdown cells, neither overexpression nor knockdown of LncPTEN1 changed migration capacity, indicating that Vimentin is indispensable for LncPTEN1-mediated metastasis suppression of lung cancer cells (Fig. 6J and K). These findings demonstrate that LncPTEN1 regulates the EMT pathway and cancer progression which depends on its interaction with Vimentin.

### 3.6 LncPTEN1 serves as scaffold to facilitate Trim16 mediated vimentin degradation

**Fig. 7:**
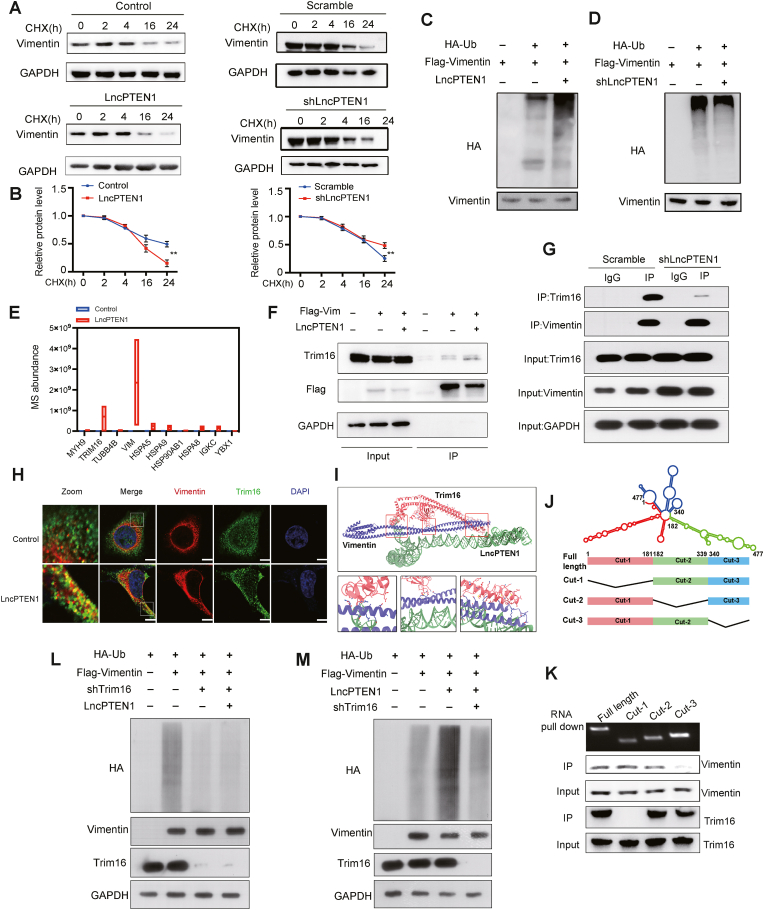
LncPTEN1 facilitates TRIM16 mediated Vimentin degradation. (A) CHX chasing assays were performed in cells with LncPTEN1 overexpression or knockdown. Western blot analysis of Vimentin protein levels after CHX treatment at the indicated times. (B) Quantification of Vimentin levels was performed using ImageJ and normalized to time 0. Data are shown as mean ± SD, ∗∗p < 0.01. (C–D) Ubiquitination assay showing Vimentin ubiquitination levels in cells with LncPTEN1 overexpression (C) or knockdown (D). (E) Mass spectrometry analysis showing potential LncPTEN1-interacting proteins. (F) Co-immunoprecipitation showing interaction between Flag-Vimentin and TRIM16 in the overexpression or knockdown of LncPTEN1. (G) Co-IP assay showing TRIM16-Vimentin interaction in scramble and LncPTEN1 knockdown cells. (H) Immunofluorescence showing colocalization of Vimentin (red) and TRIM16 (green) in control and LncPTEN1-overexpressing cells. Scale bars, 20 μm. (I) Predicted structural model of LncPTEN1-TRIM16-Vimentin interaction complex. (J) Schematic representation of LncPTEN1 truncation constructs. (K) RNA pull-down and IP assays using different LncPTEN1 truncation constructs. (L–M) Ubiquitination assay showing Vimentin ubiquitination levels in cells with TRIM16 knockdown and LncPTEN1 overexpression.

To elucidate how LncPTEN1 regulates Vimentin protein abundance, we performed cycloheximide (CHX) assays. LncPTEN1 overexpression significantly accelerated Vimentin protein degradation, while its knockdown increased Vimentin stability (Fig. 7A and B). In addition, LncPTEN1 overexpression increased the ubiquitination of Vimentin (Fig. 7C), and the opposite effect was observed in LncPTEN1 knockdown cells (Fig. 7D). Subsequently, our mass spectrometry analysis identified TRIM16, a known E3 ubiquitin ligase which was reported to promote the ubiquitination of Vimentin in lung cancer cells, as a potential LncPTEN1-interacting protein (Fig. 7E, Fig. S5A) [33], indicating that LncPTEN1 may suppress lung cancer progression by modulating TRIM16-mediated degradation of Vimentin.

Co-IP assays revealed that LncPTEN1 overexpression enhanced the association between TRIM16 and Vimentin (Fig. 7F), while LncPTEN1 knockdown weaken this interaction (Fig. 7G). Moreover, IF analysis revealed enhanced co-localization between Trim16 and vimentin upon LncPTEN1 overexpression, suggesting that LncPTEN1 may function as a molecular scaffold facilitating the interaction between these two proteins (Fig. 7H). Using AlphaFold3, we modeled the structural interfaces among LncPTEN1, Vimentin, and TRIM16 [34], the prediction revealed specific interaction surfaces and LncPTEN1 served as scaffold in this complex (Fig. 7I). We also designed LncPTEN1 truncation constructs of varying lengths based on its secondary structure [35], revealing that the N-terminal domain of LncPTEN1 binds to Trim16, while its C-terminal domain binds to Vimentin (Fig. 7J and K). LncPTEN1 overexpression led to the increase of Vimentin ubiquitination, whereas this effect disappeared in Trim16 knockdown cells demonstrating that Trim16 is essential for LncPTEN1 mediated Vimentin degradation (Fig. L,M) Collectively, these findings elucidate a molecular mechanism whereby LncPTEN1 functions as a critical scaffold to promoteTrim16-mediated Vimentin ubiquitination and subsequent proteasomal degradation, thereby attenuating EMT progression and ultimately suppressing lung cancer metastasis (Fig. S7).

## 4 Discussion

Cancer initiation and progression are driven by the complex interplay between genetic mutations and epigenetic alterations, leading to dysregulation of critical cellular pathways [36,37]. This process encompasses a series of intricate steps where cancer cells from the original tumor adapt and change, allowing them to invade nearby tissue [38,39]. LncRNAs have the capability to promote or suppress various stages of the invasion-metastasis cascade through diverse mechanisms. Further characterization of metastasis-associated lncRNA has revealed their functional roles as oncogenes, tumor suppressors, biomarker candidates, and putative therapeutic targets [40,41]. For example, the oncogenic lncRNA H19 is abnormally overexpressed in various cancer types and promotes proliferation, metastasis, therapeutic resistance, and evasion of apoptosis [42,43].

In non-small cell lung cancer, loss of PTEN protein occurs in 74 % of cases, particularly affecting well-differentiated tumors and early-stage disease [44]. Mutations and dysregulations in the PTEN/PI3K/AKT pathway significantly influence cellular processes critical for lung cancer progression, including cell survival, proliferation, and invasion. However, the exploration of the non-coding RNA landscape derived from the *PTEN* gene was previously uncharted. Through comprehensive transcriptome profiling, we identified a novel long non-coding RNA generated from the *PTEN* locus (ENST00000487939.1), designated as LncPTEN1. Expression analysis demonstrated differences in LncPTEN1 levels between lung tumor specimens and matched adjacent normal tissues, with significant downregulation observed in tumor tissues. Clinical correlation studies demonstrated that reduced LncPTEN1 expression associates with poor patient outcomes, establishing its potential as a novel prognostic biomarker in lung cancer.

N6-methyladenosine (m6A) regulates multiple aspects of RNA fate determination, encompassing alternative splicing events, transcript stability control, protein synthesis modulation [45]. The biological significance of m6A-modified transcripts is interpreted by specific reader proteins, including the YTH domain family members, which selectively recognize and bind to methylated RNA sequences [46]. Among these readers, the nuclear-localized YTHDC1 emerges as a central coordinator of RNA processing. YTHDC1 exhibits dual regulatory functions in pre-mRNA splicing: it specifically recruits the splicing enhancer SRSF3 while simultaneously preventing SRSF10 binding, thereby precisely controlling exon inclusion patterns [47,48]. This regulatory function depends on YTHDC1's m6A-binding capacity. Furthermore, YTHDC1 serves as a crucial mediator of nuclear export for m6A-modified transcripts by facilitating their interaction with the SRSF3-NXF1 export machinery, ensuring efficient nuclear-cytoplasmic trafficking of processed mRNAs [49]. In this study, we demonstrate that YTHDC1 recognizes m6A modifications in *PTEN* pre-mRNA in lung cancer, and promotes LncPTEN1 expression through enhanced splicing.

**Fig. 8:**
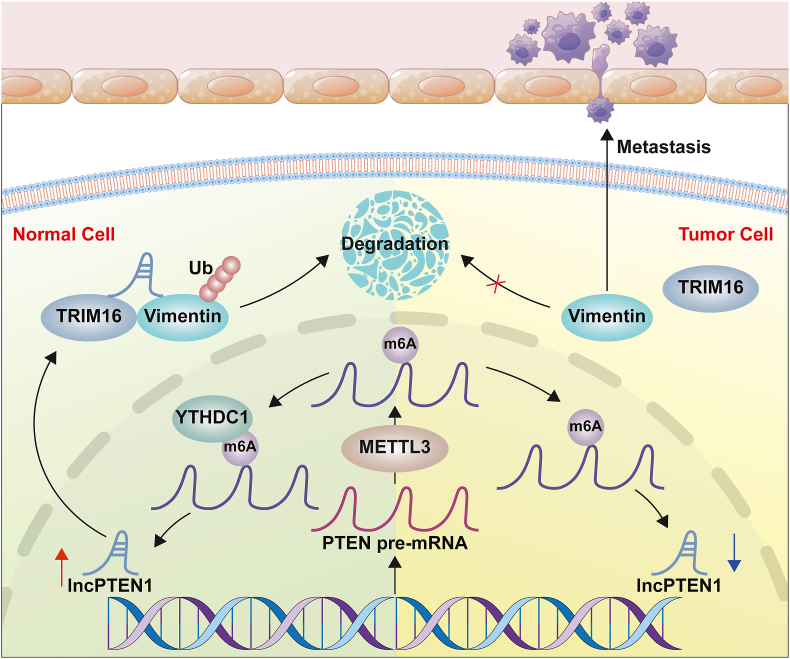
A schematic model of LncPTEN1 functions in Lung cancer cells. LncPTEN1 is a novel long non-coding RNA generated from PTEN gene and its expression is promoted by the m6A reader YTHDC1. LncPTEN1 promotes Trim16-mediated ubiquitination and degradation of Vimentin, thereby suppressing EMT progress and lung cancer invasion.

The phenotypic conversion from epithelial to mesenchymal state (EMT) represents a dynamic cellular reprogramming process characterized by dissolution of intercellular junction and acquisition of migratory capabilities. This biological transformation, regulated by key transcriptional modulators including SNAIL, TWIST and ZEB family proteins, plays an essential role in tumor metastatic colonization [6,50,51]. Vimentin is now considered a key marker for EMT, recognized for its role in the onset of the tumor metastasis cascade [52,53]. Previous studies have demonstrated that E3 ubiquitin ligases can regulate Vimentin stability and thereby influence tumor invasion [54,55]. In this study, we demonstrated that LncPTEN1 promotes ubiquitination of Vimentin mediated by Trim16 to facilitate Vimentin degradation, thereby inhibiting the EMT process and suppressing lung cancer metastasis (Fig. 8).

## 5 Conclusion

In this study, we have characterized a previously unreported long non-coding RNA, LncPTEN1, transcribed from the tumor suppressor *PTEN* gene. We demonstrate that LncPTEN1 splicing is promoted by the m6A reader protein YTHDC1, resulting in reduced LncPTEN1 expression in lung cancer cells. Moreover, low LncPTEN1 levels correlate with poor survival and increased metastasis in lung cancer, implicating LncPTEN1 as a putative prognostic biomarker for disease progression. Mechanistically, LncPTEN1 scaffolds the interaction between Trim16 E3 ligase and Vimentin, increasing Vimentin ubiquitination and proteasomal degradation, thereby suppressing EMT and metastatic progression. The identification of LncPTEN1 reveals the molecular basis of the diversity of PTEN gene function. Characterization of the tumor suppressive role of LncPTEN1 in cancer progression via modulating Trim16 mediated Vimentin degradation provided promising therapeutic target for lung cancer metastasis.

## CRediT authorship contribution statement

**Zichu Shen:** Writing – original draft, Conceptualization. **Ailin Zhong:** Formal analysis, Data curation. **Chun Zhang:** Project administration, Methodology. **Xiaowei Tang:** Data curation. **Xuyang Zhao:** Methodology. **Zhiyuan Hou:** Visualization, Methodology. **Hui Liang:** Writing – review & editing, Validation, Supervision. **Yuxin Yin:** Supervision, Funding acquisition.

## Ethics declarations

All human subject research protocols adhered to Helsinki Declaration guidelines and received approval from the Ethics Committee Board of Peking University People's Hospital (Reference: 2020PHB190-01; approved June 18, 2021). Animal experimentation procedures were conducted under protocols authorized by the Institutional Animal Care and Use Committee at Peking University Health Science Center (Protocol ID: BCJH0201; approved August 23, 2023), following Basel Declaration guidelines for animal welfare.

## Funding

Financial support was provided by the following funding agencies: 10.13039/501100012166National Key Research and Development Program of China (No. 2021YFA1300601); 10.13039/501100001809National Natural Science Foundation of China (Nos. 82273439 and 82030081); and the Systems Biomedicine Research Fund from the Lam Chung Nin Foundation.

## Declaration of competing interest

The authors declare that they have no known competing financial interests or personal relationships that could have appeared to influence the work reported in this paper.

## Abbreviations

***LUAD***: Lung adenocarcinoma
***LncRNA***: long non-coding RNA
***EMT***: Epithelial-mesenchymal transition
***RIP***: RNA immunoprecipita
***FISH***: Fluorescent in situ hybridization
***CHX***: cycloheximide
