# Supplementary material for: LncPTEN1, a long non-coding RNA generated from PTEN, suppresses lung cancer metastasis through the regulation of EMT progress
Source: Noncoding RNA Res. 2025 May 24;14:25–37. doi: 10.1016/j.ncrna.2025.05.011 (PMC12167124; doi:10.1016/j.ncrna.2025.05.011)
Supplement: Multimedia component 1 [file mmc1.docx]

**Supplementary figures**


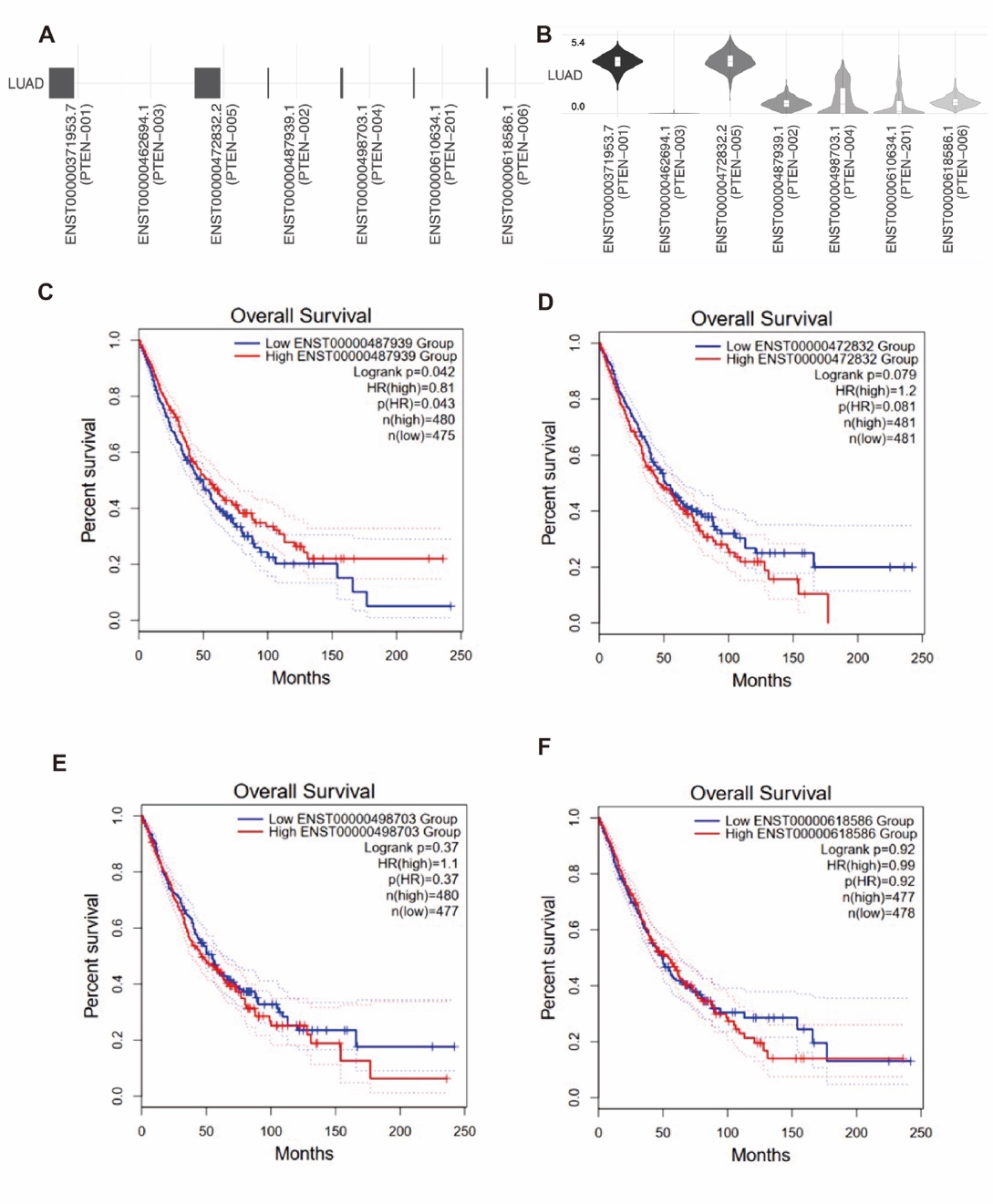


**Fig. S1. The long non-coding RNAs encoded by PTEN and their association with overall survival in lung cancer**

A-B. Expression level of PTEN-encoded lncRNAs in Lung Adenocarcinoma (LUAD)

C. Analysis of associations between lncRNA ENST00000487939 transcript levels and

overall survival in lung cancer using publicly available data on the GEPIA2.

D. Analysis of associations between lncRNA ENST00000472832 transcript levels and

overall survival in lung cancer using publicly available data on the GEPIA2.

E.Analysis of associations between lncRNA ENST00000498703 transcript levels and

overall survival in lung cancer using publicly available data on the GEPIA2.

F. Analysis of associations between lncRNA ENST00000618586 transcript levels and

overall survival in lung cancer using publicly available data on the GEPIA2.


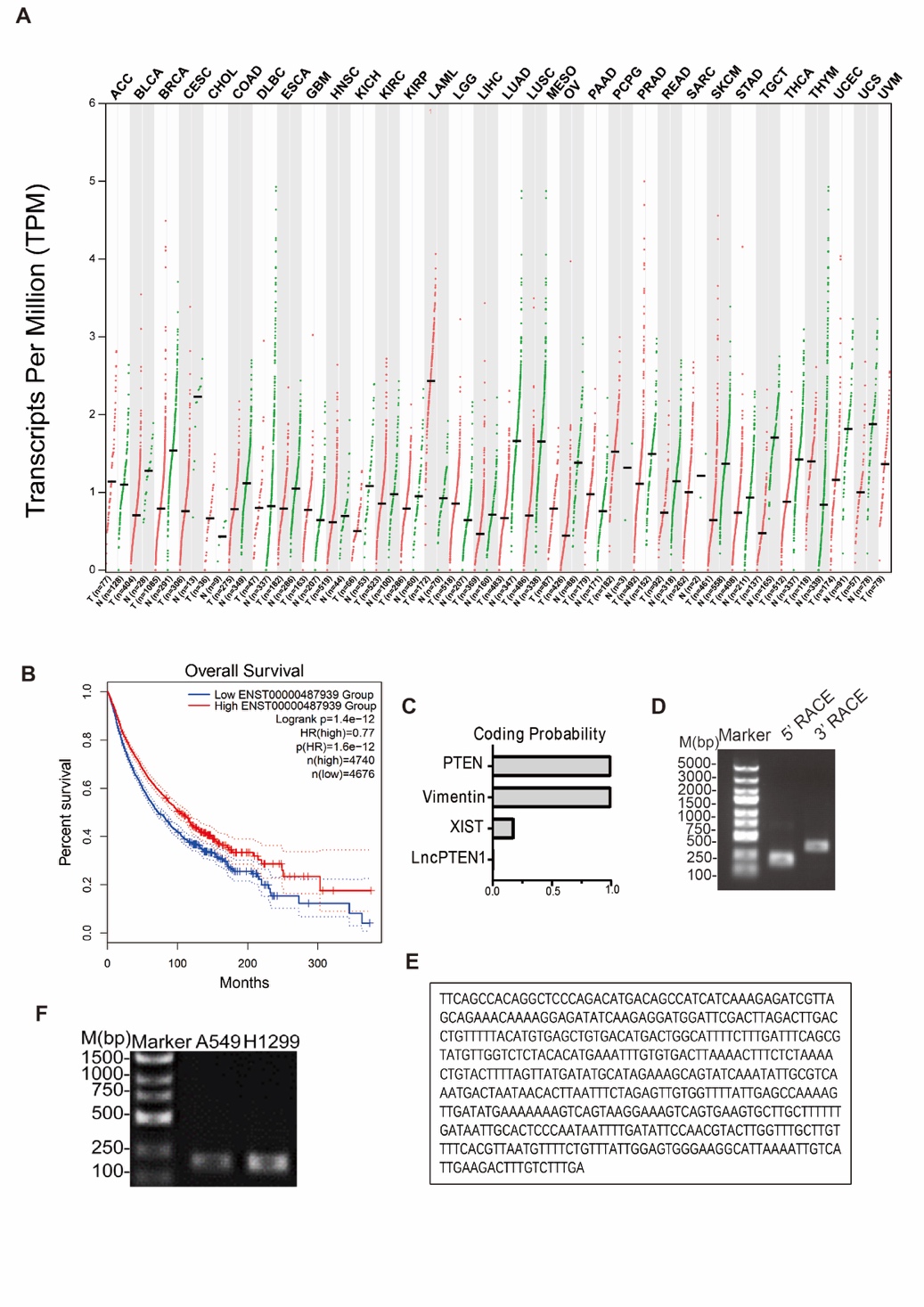


**Fig. S2. Identification and expression pattern of LncPTEN1 across cancer types**

A. Expression levels of LncPTEN1 in tumor and normal tissues across multiple cancer

types.

B. Kaplan-Meier survival analysis of LncPTEN1 expression levels in TCGA cancer database.

C. Analysis using coding potential calculator (CPC) predicted LncPTEN1 low coding probability scores.

D. 5’ and 3’ rapid amplification of cDNA ends (RACE) confirms the definitive existence of LncPTEN1.

E. The complete sequence of LncPTEN1 obtained after full-length sequencing.

F. Gel electrophoresis of qPCR products demonstrated specific amplification of LncPTEN1.


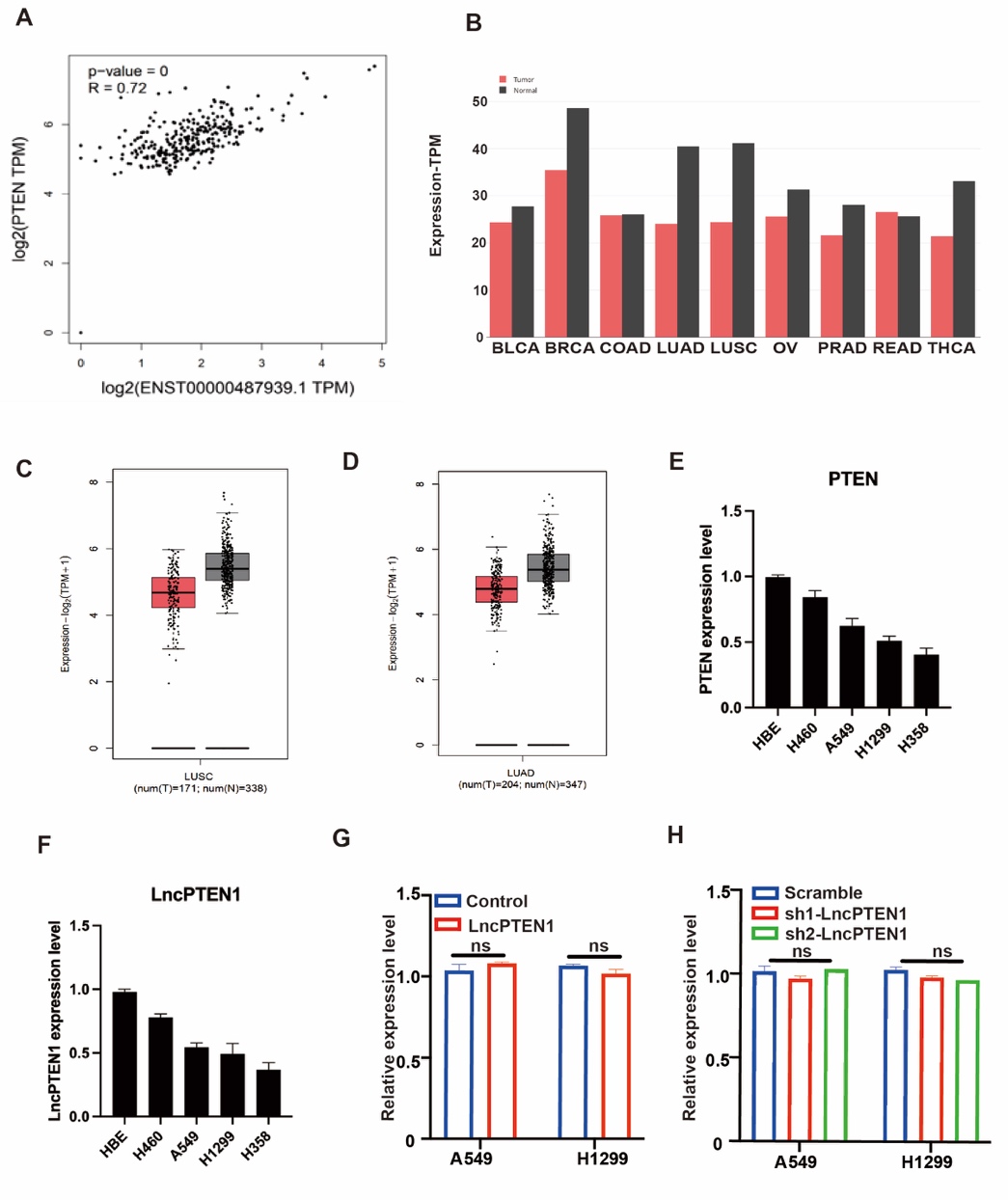


**Fig. S3. Correlation analysis of PTEN and LncPTEN1 expression**

A. The Expression correlation of PTEN and LncPTEN1 in lung cancer.

B. Expression levels of PTEN in tumor and normal tissues across multiple cancer

types.

C-D. Expression levels of PTEN in LUSC and LUAD.

E-F. Expression of PTEN and LncPTEN1 in multiple lung cancer cell lines.

G-H. Effects of LncPTEN1 overexpression and knockdown on PTEN levels in lung

cancer cell lines.


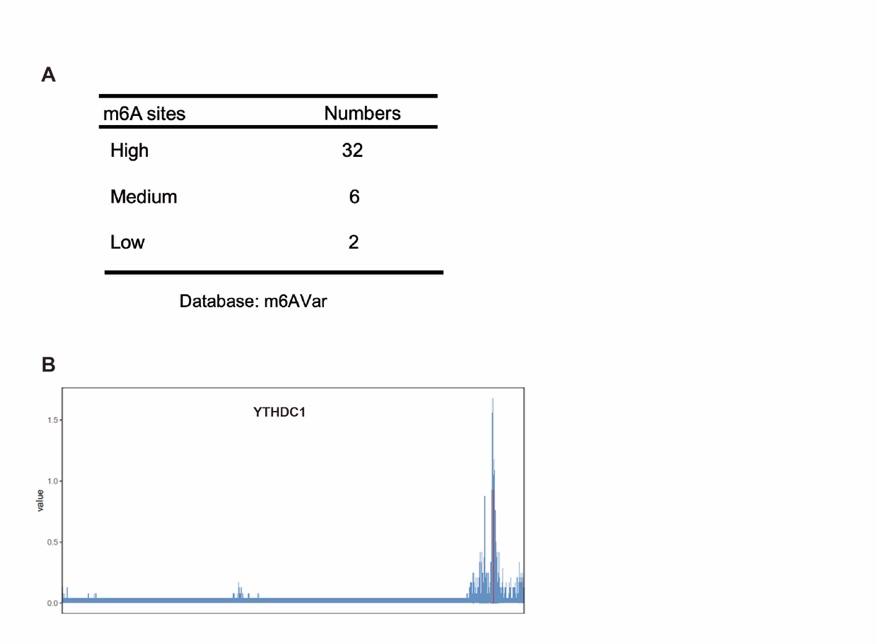

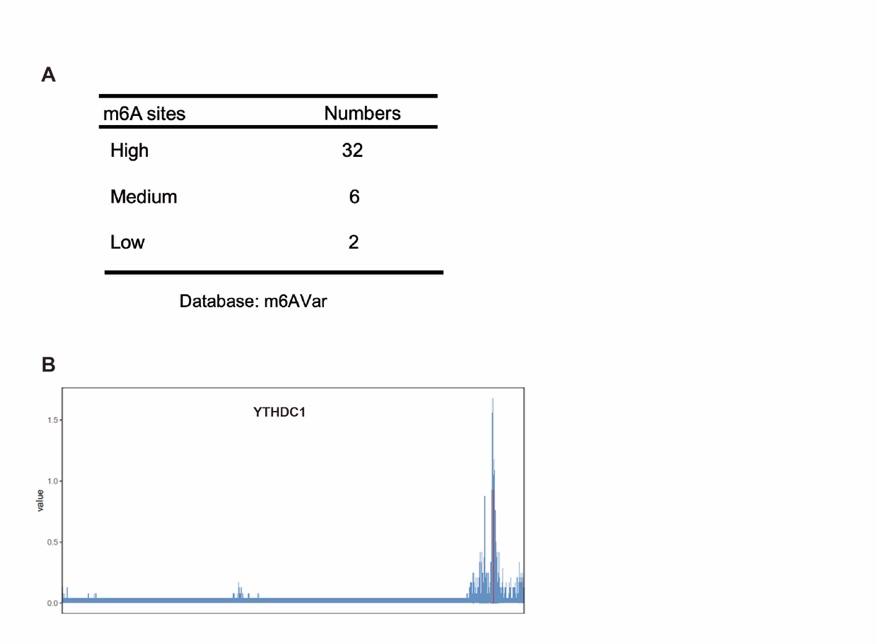


**Fig. S4. YTHDC1 recognition of m6A modification sites on PTEN mRNA**

A. Numbers of m6A modification sites on *PTEN* exon 1.

B. iCLIP peak profile of YTHDC1 in A549 cells.

**Fig. S5. LncPTEN1 functions as a novel suppressor of the EMT pathway.**

A. Western blot analysis of EMT-related transcription factors (ZEB1, E-cadherin, Slug, and SNAIL) in Scramble, shLncPTEN1 cells and shLncPTEN1 cells with rescue LncPTEN1. GAPDH was used as control.

B-C. Relative mRNA expression levels of EMT-related genes were measured by qPCR in cells with LncPTEN1 overexpression (B) or knockdown (C). Expression levels were normalized to GAPDH. Data are presented as mean ± SD from three independent experiments. **p < 0.01; ns, not significant.


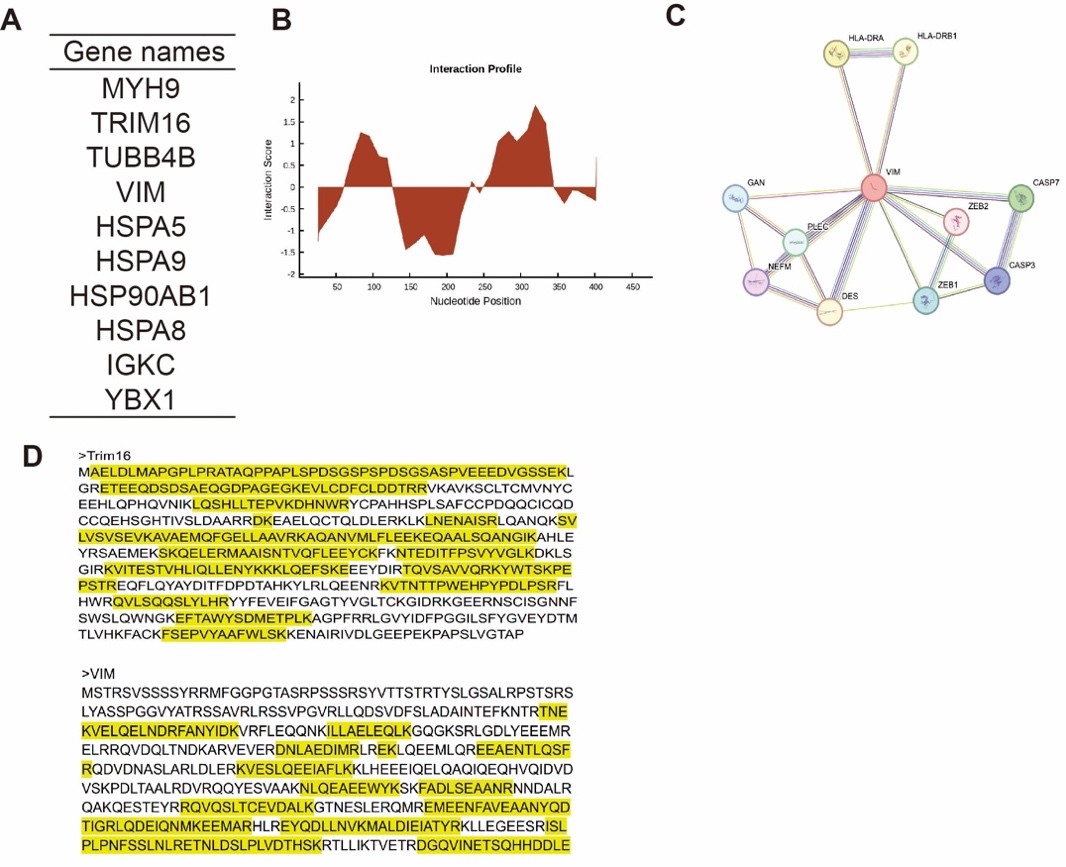


**Fig. S6. Identification and characterization of LncPTEN1 interacting proteins**

A. Top 10 proteins identified by LC-MS/MS analysis following LncPTEN1 RNA pull-down, ranked by peptide abundance and coverage. The table shows protein names, peptide counts, and fold enrichment compared to control.

B. Interaction region between LncPTEN1 and vimentin predicted by the catRAPID website.

C. Significance of Vimentin in EMT signaling pathway and its regulatory relationship with identified interaction partners.

D. Schematic representation of identified peptide fragments (highlighted in yellow) from mass spectrometry analysis, showing specific regions of Vimentin and TRIM16 that interact with LncPTEN1.

**Fig. S7. Rescue of LncPTEN1 suppresses cell migration in shLncPTEN1 cells.**

A. Representative images of transwell migration assays in Scramble, shLncPTEN1, and shLncPTEN1 cells rescued with full-length or truncated LncPTEN1 (Rescue Cut1, Cut2, Cut3).

B. Quantification of migrated cells from (A).

C. Representative images of transwell migration assays in Scramble, shLncPTEN1, shLncPTEN1 with Rescue LncPTEN1, and shLncPTEN1 with WFA (Withaferin A) cells.

D. Quantification of migrated cells from (C).Cell numbers were counted from at least three random fields per well. Data are presented as mean ± SD from three independent experiments. ***p < 0.001; ns, not significant.

**Supplementary Table 1. A list of antibodies used in this study**

| Species | Antigen | Company(Cat#) | Dilution ratio |
| --- | --- | --- | --- |
| Mouse monoclonal | Vimentin | Santa Cruz (sc-6260) | 1:1000 WB |
| Mouse monoclonal | GAPDH | Sungene Biotech(KM9002) | 1:5000 WB |
| Mouse monoclonal | FLAG | Sigma-Aldrich (F3165) | 1:5000 WB |
| Mouse monoclonal | HA | Sigma-Aldrich (H9658) | 1:5000 WB |
| Rabbit monoclonal | Trim16 | Abcam(ab72129) | 1:2000 WB |
| Mouse monoclonal | ZEB1 | Santa Cruz (sc-515797) | 1:1000 WB |
| Mouse monoclonal | E-cadherin | Santa Cruz (sc-8426) | 1:1000 WB |
| Rabbit monoclonal | SLUA | Abcam(ab302780) | 1:5000 WB |
| Rabbit monoclonal | SNAIL | Abcam(ab216347) | 1:5000 WB |

**Supplementary Table 2. Oligonucleotides sequences**

| **Gene** | **Sequence** |
| --- | --- |
| GAPDH-F | GGAGCGAGATCCCTCCAAAAT |
| GAPDH-R | GGCTGTTGTCATACTTCTCATGG |
| ACTB-F | CATGTACGTTGCTATCCAGGC |
| ACTB-R | CTCCTTAATGTCACGCACGAT |
| PTEN-F | TGGATTCGACTTAGACTTGACCT |
| PTEN-R | GGTGGGTTATGGTCTTCAAAAGG |
| VIM-F | GACGCCATCAACACCGAGTT |
| VIM-R  Trim16-F  Trim16-R | CTTTGTCGTTGGTTAGCTGGT  GTCCTGTCTAACCTGCATGGT  GGCAGTATCGCCAGTTGTG |
| U1-F | TCCCAGGGCGAGGCTTATCCATT |
| U1-R | GAACGCAGTCCCCCACTACCACAAAT |
| ZEB1-F | CAGCTTGATACCTGTGAATGGG |
| ZEB1-R | TATCTGTGGTCGTGTGGGACT |
| Slug-F | TGTGACAAGGAATATGTGAGCC |
| Slug-R | TGAGCCCTCAGATTTGACCTG |
| N-cadherin-F | AGCCAACCTTAACTGAGGAGT |
| N-cadherin-R | GGCAAGTTGATTGGAGGGATG |
| Smad2-F | TCATAGCTTGGATTTACAGCCAG |
| Smad2-R | TTCTACCGTGGCATTTCGGTT |
| ZNF703-F | CCCTCCAGCATTGGCTACC |
| ZNF703-R | CAATAGGGGTCGCGGCATAAT |
| LncPTEN1-PCR-F | TTCAGCCACAGGCTCCCAGAC |
| LncPTEN1-PCR-R | TCAAAGACAAAGTCTTCAATGACAATTTTAATGC |
| LncPTEN1-qP-F | CTTAGACTTGACCTGTTTTTACATGT |
| LncPTEN1-qP-R | TCAAAGACAAAGTCTTCAATGACAATT |
|  |  |
| LncPTEN1 sense | CACAGCTCACATGTAAAAACAGGTCAAGTCTAAGTCGAAT |
| LncPTEN1 antisense | ATTCGACTTAGACTTGACCTGTTTTTACATGTGAGCTGTG |
| LncPTEN1-3'CY3 | CACAGCTCACATGTAAAAACAGGTCAAGTCTAAGTCGAAT-CY3 |
|  |  |
| SH1-YTHDC1 | TGGATTTGCAGGCGTGAATTA |
| SH2-YTHDC1 | TGCCTCCAGAGAACCTTATAA |
| SH1-Vimentin | GCCTTAACATTGCCCACTGAT |
| SH2-Vimentin  SH1-Trim16  SH2-Trim16 | GCAAGTATGTTCACTATGAAA  GCCGTTGTTCAGCGCAAATAT  CCGCATCAGGTGAACATCAAA |
| SH1-LncPTEN1 | GACTTAGACTTGACCTGTTTTT |
| SH2-LncPTEN1 | AGACTTGACCTGTTTTTACATG |
